# B-Mode Ultrasound May Be an Early Marker in Acute Kidney Injury

**DOI:** 10.3390/diagnostics15162034

**Published:** 2025-08-14

**Authors:** André Luiz Sampaio Fernandes, Fernanda Gosuen Gonçalves Dias, Marcela Aldrovani Rodrigues, Ewaldo de Mattos-Junior, Alef Winter Alvarenga, Maria Eduarda Raffaini de Oliveira Cunha, Marjury Cristina Maronesi, Leandro Zuccolotto Crivellenti

**Affiliations:** 1 Programa de Pós-Graduação em Ciência Animal, Universidade de Franca, Franca 14404-600, SP, Brazil; andre.sampaio94@hotmail.com (A.L.S.F.); fernanda.dias@unifran.edu.br (F.G.G.D.); marcela.rodrigues@unifran.edu.br (M.A.R.); ewaldomattos@hotmail.com (E.d.M.-J.); alefwinter@hotmail.com (A.W.A.); 2 Programa de Pós Graduação em Ciência Veterinárias, Universidade Federal de Uberlândia, Uberlândia 38400-902, MG, Brazil; crivellenti_lz@yahoo.com.br; 3 Diagnóstico Vet—Ultrassonografia Veterinária, Barretos 14783-104, SP, Brazil; marjury_mah@hotmail.com

**Keywords:** compression elastography, Doppler, nephrotoxicity, resistance index

## Abstract

**Background/Objectives:** This study evaluated the applicability of B-mode ultrasound, Doppler, and elastography in the early diagnosis of non-azotemic acute kidney injury (AKI) in rats induced with cyclophosphamide. **Methods:** The prospective, randomized, and blinded experiment involved groups receiving cyclophosphamide (CG, *n* = 12) and saline (control, SG, *n* = 9). Serum biomarkers (urea, creatinine, and symmetric dimethylarginine) were assessed, along with renal histological analysis to classify AKI severity and distribution. **Results:** B-mode ultrasound revealed a significantly higher corticomedullary ratio at 24 and 72 h and increased renal width at 48 h in the cyclophosphamide group compared to controls. Biochemical analyses showed no significant differences between groups in early stages. Although B-mode ultrasound detected early morphological changes—specifically in corticomedullary ratio and renal size—Doppler and elastography demonstrated limited diagnostic utility in early AKI detection. **Conclusions:** Overall, B-mode ultrasound provided valuable early indicators of renal injury, whereas Doppler and elastography showed minimal clinical benefit at this stage.

## 1. Introduction

The kidneys play a crucial role in maintaining the overall balance of an organism by eliminating metabolic waste products and detoxifying substances and toxins [1]. However, these essential organs are vulnerable to damage caused by various agents, including nephrotoxic chemotherapeutic drugs [2]. Furthermore, renal ischemia is a cause of AKI, in which early detection is important [3]. AKI is characterized by a rapid decline in renal function and structural integrity [4,5]. This deterioration may manifest as elevated creatinine and urea levels, with or without oliguria [6,7,8,9,10,11].

The incidence of AKI among hospitalized individuals receiving oncological care is estimated to be around 12%, with injury often occurring within the first 48 h of admission [12,13,14,15]. Interestingly, in intensive care units specialized in oncology, this incidence can rise as high as 50% [16].

Identifying and managing disease progression at an early stage pose significant challenges, as conventional laboratory biomarkers have limited sensitivity in detecting renal damage during the initial phases [17,18]. Additionally, creatinine is a late biomarker, and its levels require adjustments for risk factors such as ethnicity, age, and gender, which can influence the interpretation of results [17].

Although renal biopsy is currently the most accurate method for evaluating the progression of renal disorders, its invasiveness, potential sampling errors, and challenges in longitudinal monitoring impose significant limitations [19,20,21]. Therefore, there is a critical need to explore non-invasive and reproducible alternatives. Imaging techniques have emerged as an area of investigation for assessing both AKI and chronic kidney disease (CKD) [22,23,24,25]. Ultrasonography is commonly used as the initial imaging modality [23,26,27,28], while Doppler is employed specifically when renal vascular abnormalities are suspected [29,30,31]. However, these methods have inherent limitations in their ability to provide comprehensive and quantifiable information on renal morphology/echogenicity and hemodynamics [25].

Recent advances include three ultrasound elastography techniques—strain elastography, acoustic radiation force impulse elastography (ARFI), and point shear wave elastography (p-SWE) [32,33,34]. These modalities have emerged as promising noninvasive methods for the assessment of renal impairment [24,25,34,35,36,37] and the detection of early stages of CKD [25,36]. However, its application in AKI is still limited [35], with few studies correlating elastography findings with the extent of histopathological injury in AKI, in addition to the lack of systematic comparisons evaluating the diagnostic performance of elastography in relation to conventional ultrasound techniques in AKI [36].

Given the lack of minimally invasive early markers and the limited availability of studies employing a multimodal ultrasound approach—including B-mode ultrasound, Doppler, and strain elastography—in the detection of acute kidney injury (AKI), this study aims to identify AKI early in rats with cyclophosphamide-induced nephrotoxicity using these imaging techniques.

## 2. Material and Methods

### 2.1. Animals

This study was conducted in accordance with the guidelines and approval of the Animal Ethics Committee of the University of Franca (CEUA/UNIFRAN, protocol number 6168260121).

A total of 21 male non-castrated Wistar rats (*Rattus norvegicus*) aged between 10 to 30 weeks, weighing 150–300 g, were used in this study. Restriction of the sex of the animals was based on an attempt to minimize the influence of hormonal variations, which can occur in females, even considering relatively short experimental periods. Regarding age, we opted for young animals to reduce possible renal interferences from pre-existing factors. The rats were in a healthy condition and underwent a seven-day acclimatization period to adapt to the experimental conditions.

In the experimental phase, the rats were housed individually in polypropylene cages that provided adequate ventilation, a controlled temperature of 22 ± 2 °C, humidity levels of 50 ± 10%, and a 12 h light–dark cycle. Additionally, they received environmental enrichment with paper rolls and cotton wool. The rats had unrestricted access to drinking water and were fed a standard commercial diet for rodents (Presence Rat and Mouse Diet—Animal Nutrition, Paulínia, SP, Brazil).

### 2.2. Experimental Design

The animals were randomly distributed into two groups: the cyclophosphamide group (CG, *n* = 12) and the saline group (control) (SG, *n* = 9), using the randomization tool (www.randomizer.org). The CG group received a single intraperitoneal injection of cyclophosphamide (Sigma-Aldrich Ltda., São Paulo, SP, Brazil) at a dose of 150 mg/kg [38]. The GS group received a single intraperitoneal injection of 150 mL/kg of 0.9% NaCl solution. Experimental evaluation was performed 24, 48, and 72 h after injection. Only one operator, who was blinded to the treatment, evaluated the clinical signs.

### 2.3. Renal Ultrasonographic Examinations

**Figure 1:**
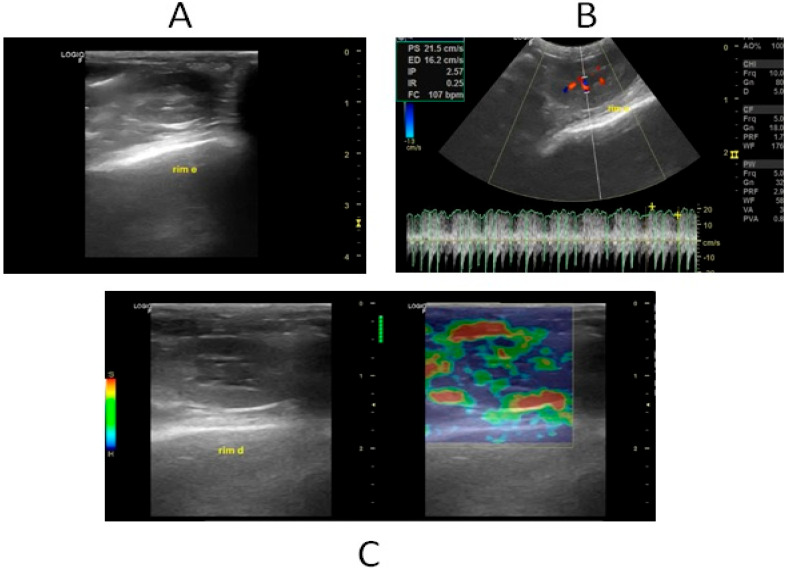
B-mode ultrasound, Doppler, and basal renal elastography. (**A**) Renal B-mode ultrasound demonstrating a border with regular contours, uniform echotexture, echogenicity, and a normal corticomedullary relationship. (**B**) Renal Doppler evaluation demonstrating renal artery resistivity index within normal limits. (**C**) Renal strain elastography demonstrating variable colorimetric pattern, but with more pronounced blue areas in the cortex and green areas in the medulla, reflecting high stiffness in the cortex and low stiffness in the medulla.

Renal ultrasonographic examinations (B-mode, Doppler, and strain elastography) (Figure 1A–C) were conducted on both groups after 24, 48, and 72 h.

During each experimental time point, four animals from the CG group and three from the GS group were included in the evaluation. Before the examinations, the animals were anesthetized using a single intraperitoneal application of a combination of 10% ketamine hydrochloride (50 mg/kg, Agener União Saúde Animal, Embu-Guaçu, SP, Brazil) and 2% xylazine hydrochloride (5 mg/kg, Konig, Mairinque, SP, Brazil).

After the trichotomy of the entire abdominal area, the animals were positioned in the dorsal and lateral decubitus to assess the right and left kidneys using longitudinal and transverse scans. The examinations were conducted using a GE LOGIQ F6 ultrasound device (GE General Electric, São Paulo, SP, Brazil) equipped with a 7.75 MHz matrix and multifrequency linear transducer, as well as a 6.5 MHz microconvex transducer. Before each examination, ultrasound gel was applied locally.

### 2.4. B-Mode Ultrasound

Renal structures were evaluated using the B-mode. The scoring criteria used for the assessment are presented in Table 1.

The corticomedullary ratio was calculated by measuring the cortex and medulla in the longitudinal section, and it was deemed normal when the ratio was one-to-one. Additionally, the ratio of renal length to aortic diameter was determined using the maximum renal length and the maximum diameter of the aortic lumen, excluding the wall at the level of the kidneys.

### 2.5. Doppler Ultrasound

Doppler ultrasound was utilized to examine the renal artery and interlobar, arcuate, and interlobular arterioles. Precise location of the renal artery was ensured, and meticulous adjustments were made to achieve a Doppler beam angle of less than 60° relative to the long axis of the vessel [35].

For each assessment, three consecutive waveforms were thoroughly analyzed. The following parameters were evaluated: systolic velocity (SV in cm/s), diastolic velocity (DV in cm/s), resistive index (RI = (Vmax in cm/s − Vmin in cm/s)/(Vmax in cm/s)), and blood flow pattern categorized as low, intermediate, or high resistance, assigned corresponding scores of 1, 2, and 3, respectively.

### 2.6. Strain Elastography

Strain elastography was performed by applying manual compression to the body surface near the renal regions using an ultrasound transducer while simultaneously acquiring B-mode ultrasound images to generate color-coded elastograms depicting tissue deformation.

This study adopted scoring criteria that were adapted from a previous study [39]. The assigned scores ranged from 1 to 4. A score of 1 represented green areas in both the renal cortex and medulla, indicating low tissue rigidity. A score of 2 indicated predominantly blue areas in the cortex and green areas in the medulla, reflecting high rigidity in the cortex and low rigidity in the medulla. A score of 3 denoted green areas in the cortex and blue areas in the medulla, indicating low rigidity in the cortex and high rigidity in the medulla. Lastly, a score of 4 depicted both the cortex and medulla as blue areas, representing high tissue rigidity in the elastogram. Three observers performed the elastography scoring.

### 2.7. Creatinine, Urea, and SDMA Measurements

Following the ultrasonographic examinations, blood samples were collected from the CG and GS groups and transferred to vacuum tubes without anticoagulant. The biochemical analysis included the measurement of creatinine using the alkaline picrate reaction, urea using the urease kinetic method, and SDMA using the IDEXX test (Technology and diagnostic solutions company for Veterinary Medicine).

### 2.8. Histopathological Evaluation

After blood collection, animals from both groups were euthanized with sodium pentobarbital (Cristália Produtos Químicos e Farmacêuticos Ltd., São Paulo, SP, Brazil) at a dose of 120 mg/kg intraperitoneally, followed by histopathological analysis.

The right and left kidneys were harvested, fixed in 10% neutral buffered formalin, and then processed for routine paraffin embedding. Subsequently, 3 μm thick sections were obtained and stained with hematoxylin and eosin. The kidneys were classified into different grades of acute tubular injury. Grade 0 represented normal renal morphology, while grade 1 indicated the presence of rare tubules with evidence of acute tubular injury observed in the cortex. Grade 2 denoted small clusters of tubules with acute tubular injury, discontinuously distributed in the renal cortex. Grade 3 reflected clusters of tubules with signs of acute tubular injury frequently found in the renal cortex. Lastly, grade 4 characterized extensive areas with tubules showing signs of acute tubular injury distributed throughout the renal cortex.

### 2.9. Data Analysis

All data were analyzed using the Shapiro–Wilk test with a 5% significance level to assess residual normality and the Bartlett test with a 5% significance level to assess homogeneity of variance.

To analyze the data, a generalized linear model with a random effect was employed. This allowed for the comparison of groups and the assessment of time–response changes for all variables. A multinomial distribution was assumed for the data, and a cumulative logit link function was used to model the relationship between the predictors and the response. In cases where multiple comparisons were needed, the Sidak post hoc test was applied to adjust the significance levels. Statistical significance was considered at a *p*-value less than 0.05.

For each experiment conducted, including independent replications, the data were analyzed to provide summary and descriptive statistics for each experimental group.

All data analyses and statistical procedures were conducted using GraphPad Prism 8.0 software (GraphPad Company, San Diego, CA, USA).

## 3. Results

### 3.1. B-Mode Ultrasound

**Figure 2:**
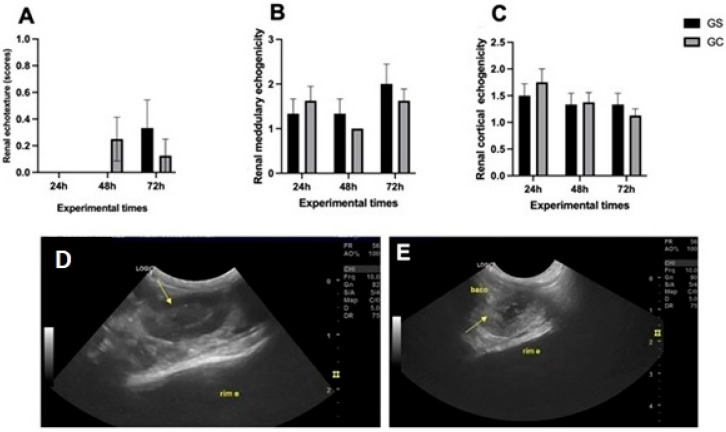
B-mode ultrasound parameters regarding echogenicity and renal echotexture. Graphical representations of B-mode ultrasonographic parameters regarding renal echogenicity and echotexture in Wistar rats after 24, 48, and 72 h of intraperitoneal administration of cyclophosphamide (GC, *n* = 12) or saline solution (GS, *n* = 9). (**A**) Renal echotexture. (**B**) Renal medullary echogenicity (score 1: anechoic, score 2: hypoechoic, score 3: hyperechoic). (**C**) Renal cortex echogenicity (score 1: < spleen or liver, score 2: equal to spleen or liver, score 3: greater than spleen or liver). (**D**) B-mode ultrasonographic parameter, demonstrating medullary hyperechogenicity, as indicated by the arrow. (**E**) B-mode ultrasonographic parameter, demonstrating cortical hyperechogenicity of the left kidney, as indicated by the arrow, of an animal treated with cyclophosphamide. There were no statistical differences between the groups at the respective experimental time points.

No significant differences (*p* > 0.05) were found between the CG and GS groups in the evaluation of renal echotexture (Figure 2A). Medullary hyperechogenicity (*p* < 0.05) was observed in three animals (25%) in the CG group and in four animals (44.4%) in the GS group (Figure 2B,D). Additionally, the CG group showed medullary hypoechogenicity in three animals (25%) and cortical hyperechogenicity (Figure 2C,E) in one animal (8.3%) compared to the echogenicity of the liver and spleen (reference standards).

**Figure 3:**
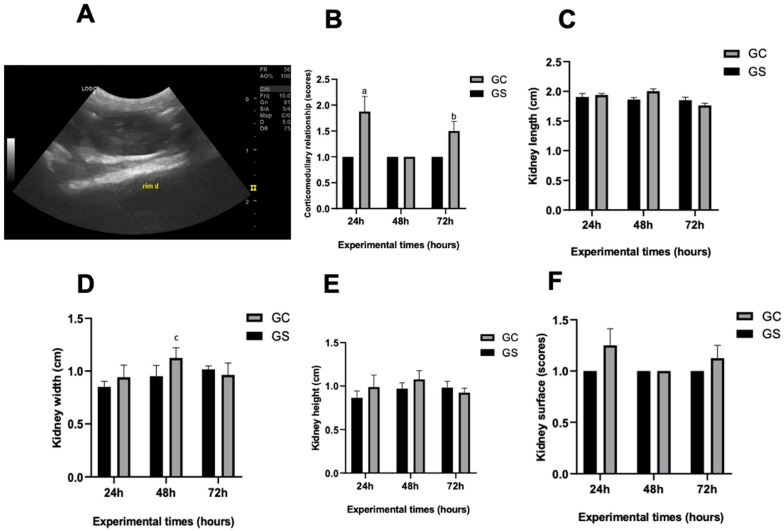
B-mode ultrasound and corticomedullary changes, size, and renal surface. (**A**) B-mode ultrasonographic parameter, demonstrating loss of corticomedullary definition of the right kidney of an animal treated with cyclophosphamide (GC). (**B**) Graphical representation of B-mode ultrasonographic parameters regarding the renal corticomedullary relationship (score 1: normal, score 2: decreased and score 3: increased). (**C**–**E**) Graphical representation of B-mode ultrasound parameter regarding renal size (cm). In (**C**), kidney length; in (**D**), renal height; and in (**E**), renal width. (**F**) Graphical representation of B-mode ultrasonographic parameter regarding renal surface (score 1: regular and score 2: irregular). ^a^ Significantly different from GS at the 24 h experimental time point. ^b^ Significantly different from GS at the 72 h experimental time point. ^c^ Significantly different from GS at the 48 h experimental time point.

The corticomedullary ratio (Figure 3A,B) showed a significant increase (*p* < 0.05) in the CG at 24 and 72 h compared to the SG. Renal size (Figure 3C–E) exhibited a significant increase only in the width of the CG at 48 h (*p* < 0.05) compared to the SG. However, no changes were observed in the ratio renal size to the abdominal aorta. Irregular renal surfaces were observed in two animals (16.6%) in the CG, but no significant differences (*p* > 0.05) were found between the groups (Figure 3F).

### 3.2. Doppler Ultrasound

**Figure 4:**
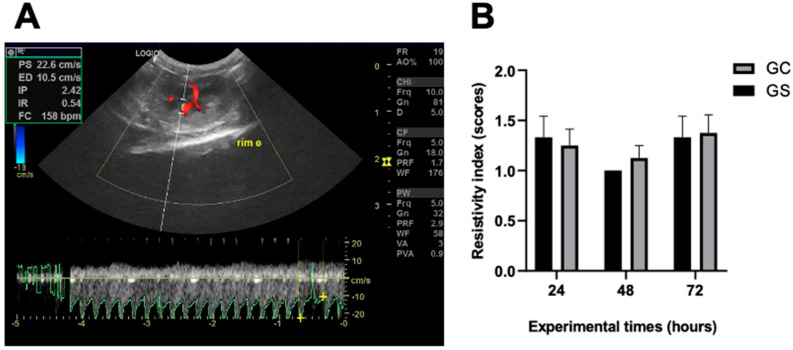
Renal resistivity index. (**A**) Doppler ultrasonographic parameter revealing intact renal vasculature and resistivity index of the left kidney of an animal treated with cyclophosphamide (GC). (**B**) Graphical representation of Doppler ultrasonographic parameter regarding renal resistive index (score 1: low, score 2: intermediate, and score 3: high). PS: systolic speed; ED: diastolic velocity; PI: pulsatility index; IR: resistivity index; HR: heart rate. There were no statistical differences between the groups in the respective experimental moments.

Doppler analysis revealed intact renal vasculature (Figure 4A), allowing for visualization and measurement in all animals regardless of the experimental time points. Regarding the renal resistive index and the pattern of renal blood flow (Figure 4B and Supplementary Material File S1), no significant differences (*p* > 0.05) were found between the groups.

### 3.3. Strain Elastography

**Figure 5:**
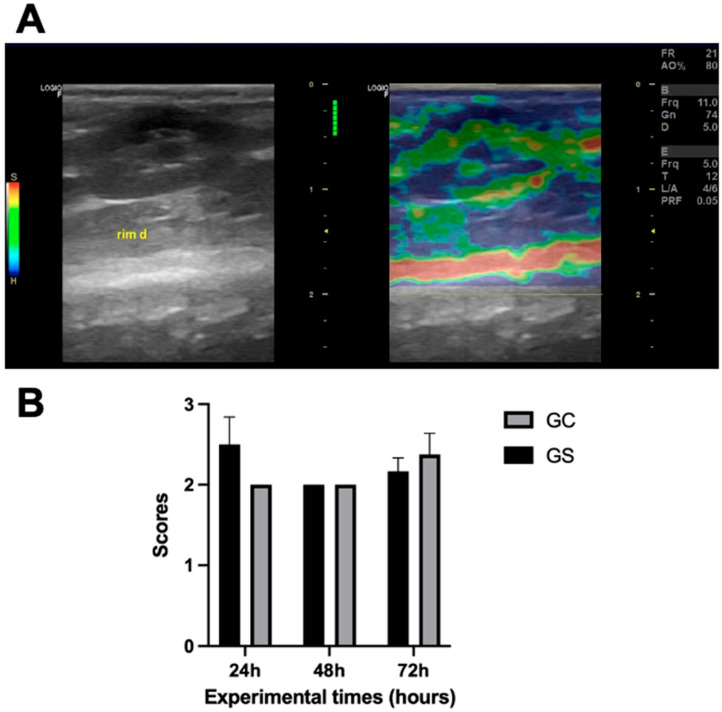
Renal strain elastography. (**A**) Image of strain elastography of the right kidney of an animal treated with cyclophosphamide (GC), demonstrating predominantly blue areas in the cortex and green areas in the medulla, reflecting high rigidity in the cortex and low rigidity in the medulla. (**B**) Graphical representation of strain elastography (score 1: low, score 2: intermediate, and score 3: high) in Wistar rats after 24, 48, and 72 h of intraperitoneal administration of cyclophosphamide (GC, *n* = 12) or saline solution (GS, *n* = 9).

No differences (*p* < 0.05) were found in strain elastography between the groups. The predominant finding in both groups was a score of 2 (Figure 5), present in all animals in the CG GS groups, in at least one evaluated kidney. A score of 3 was observed in three (25%) CG animals and two (22.2%) GS animals, while a score of 4 was seen in one (11.1%) GS animal, in at least one kidney.

### 3.4. Creatinine, Urea, and SDMA

No alterations in creatinine or urea levels were found (Supplementary Material File S2). However, SDMA analysis revealed increased levels in four animals (33.3%) in the CG group. Three of them (25%) demonstrated higher levels 24 h post-cyclophosphamide and one (8.3%) after 72 h. In the GS group, only one animal (11.1%) showed a slight increase after 72 h.

### 3.5. Histopathology

The histopathological results showed varying degrees of AKI at all experimental time points in the CG, with grade 1 observed in two animals at 48 h and grade 3 in two animals at 24 and 72 h, with changes such as tubular dilation, thinning of tubular epithelium, cylinders, cellular swelling, loss of microvilli, cell detachment, mitosis, and binucleation, as well as interstitial inflammation.

**Figure 6:**
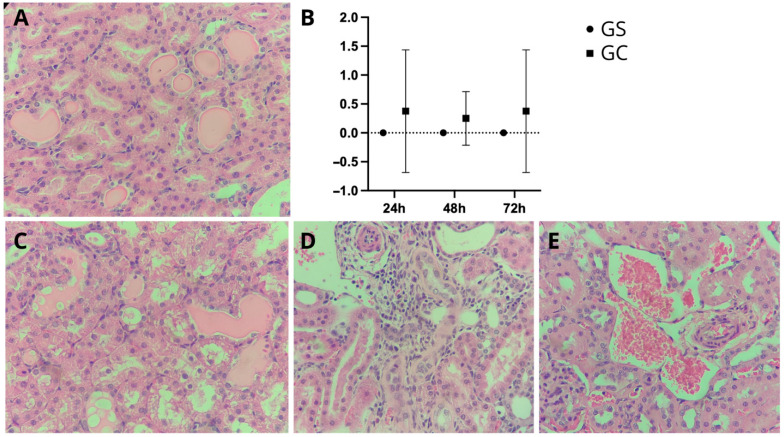
Renal histopathological analysis. Magnification 100×. Histopathological image exhibits cylindrical tubular dilatation in the kidney (**A**), scored from 0 to 4 (**B**). This dilation is evident in animals treated with cyclophosphamide (GC) but not in those treated with saline. In the GC group, cylindrical tubular dilatation (**C**) is accompanied by hydropic degeneration, interstitial inflammatory infiltrates (**D**), and congestion (**E**).

The aim of this analysis was to elucidate the effects of cyclophosphamide and saline treatments at three temporal points, with a specific emphasis on tubular degeneration (Figure 6A–C). While observable trends emerged within each group, rigorous statistical analysis demonstrated no statistically significant differences (*p* > 0.05). At the 24 h mark, the CG exhibited elevated scores of tubular degenerations in comparison to the GS, suggesting a potential early influence on this specific pathology. This trend persisted at the 72 h assessment, indicating a sustained impact of cyclophosphamide on tubular degeneration over time.

In addition to tubular degeneration, the CG group manifested discrete focal lymphoplasmacytic interstitial inflammatory infiltrates (Figure 6D) and areas of congestion (Figure 6E).

## 4. Discussion

In this study, we found that the parameters of kidney size and corticomedullary ratio based on B-mode ultrasound demonstrated significant changes in animals subjected to nephrotoxic doses of cyclophosphamide; that is, the larger the kidney size, the greater the chances of developing AKI. This is relevant as an early marker of this condition, and it may be a low-invasive and easily applicable tool for the monitoring and early diagnosis of patients at risk of developing AKI, before changes in biochemical markers such as urea, creatinine, and SDMA. Although not statistically significant, renal surface irregularities were observed, and this morphological feature may be associated with early actions of acute kidney injury. One study found that irregular renal shape was significantly more prevalent in azotemic cats (29%) compared to non-azotemic cats (5%). Although the study focused on azotemia rather than early AKI, the increased frequency of surface irregularities in affected animals supports the hypothesis that such morphological changes may reflect underlying renal pathology, even at an early or subclinical stage [40]. This suggests that this parameter deserves to be investigated in future studies, considering its potential as an early complementary marker in the ultrasonographic evaluation of AKI. On the other hand, Doppler ultrasound and strain elastography did not show significant differences, proving to be inferior to B-mode ultrasound in this study.

This study is innovative in using combined ultrasound techniques—B-mode, Doppler, and strain elastography—to assess cyclophosphamide-induced acute renal failure. Monitoring increased renal volume, especially in the cortex, appears to be a useful parameter that could be applied in both humans and animals with AKI. Previous studies in dogs have shown that increased kidney size is a key early sign of septic AKI, which aligns with our findings, where affected animals exhibited these changes on B-mode ultrasound [38]. Our results suggest that increases in renal width might occur earlier than changes in length or ratios, indicating early kidney enlargement. Future research should explore whether width could be the first parameter to change in early AKI in animals.

A recent study demonstrated that a kidney/aorta ratio > 5.93 was correlated with AKI in female dogs with sepsis and pyometra [38]. In the present study, this parameter was not significantly different between the groups. However, it is important to note that the diagnosis of AKI in pyometra occurs late, when abnormalities in biochemical and urinary markers of renal function or injury are already observed [41]. Additionally, there is an association of factors related to septicemia, causing acute renal failure [42]. Therefore, it is believed that there was no increase in the kidney/aorta ratio due to the short experimental period proposed here, since the increase in kidney size was identified early.

Additionally, although infrequent (*n* = 1, 8.3%), hyper echogenicity of the renal cortex was observed in one animal with histological grade 3 acute tubular injury, and this increase in echogenicity in this renal region may be related to acute renal injury cases [43].

The medullary hyperechogenicity (medullary sign) present in 25% of the animals in the CG group and 44.4% in the GS group was not related to acute tubular injury, indicating that, similar to humans, it should not be used as a morphological alteration in AKI [44].

One parameter that showed relevance in terms of the early identification of acute tubular injury was the reduction in corticomedullary definition. It is worth emphasizing that no animal in the saline group (control) showed alterations in this parameter. This alteration is probably due to renal degeneration and inflammation, similar to what was found in the histopathological examination demonstrated in dogs [37]. Although not the focus of this research, it can be inferred that this early alteration is likely to persist, as a similar alteration was observed in cats with AKI, and there was also a correlation of this alteration with the degree of renal functionality based on serum creatinine levels [40].

A corticomedullary trend was observed in the CG, showing an increase at 24 h, a decrease at 48 h, and an increase at 72 h (Figure 3B). These results are consistent with those presented in the histology, in which some animals in the CG group, at the 24 and 72 h periods, demonstrated elevated scores of tubular degeneration when compared to 48 h and to the SG group. These findings may be related to the ultrasound changes in the corticomedullary relationship with the histological degree of renal injury presented.

The present study did not demonstrate changes related to the integrity of the renal vessels and the resistivity index between the GC and GS groups at different experimental times. These data are relevant, since scientific studies still present discrepant results on renal hemodynamic behavior in different pathological contexts. In patients with sepsis-associated AKI, for example, no changes in these parameters were observed [38], suggesting that in certain etiologies of AKI, such as sepsis, Doppler indices may remain unchanged. On the other hand, a recent study demonstrated that the resistivity index was a significant predictor of AKI in humans at admission to the intensive care unit [45], indicating that the usefulness of this parameter may vary according to the causative agent and the time of assessment.

It is essential to understand that renal injury can occur in different compartments of the nephron—tubules, glomeruli, interstitium, and blood vessels—which directly influences ultrasound findings [46]. In the specific context of this study, the absence of changes in vascular parameters can be explained by the mechanism of action of cyclophosphamide, whose metabolites exert direct toxicity on the collecting tubules, without necessarily compromising renal hemodynamics [47]. Furthermore, autoregulatory mechanisms of renal blood flow can initially compensate for the reduction in perfusion, preserving tubular and glomerular function in the early stages of injury [46].

It should be considered that not every patient with renal injury will demonstrate alterations in renal hemodynamic indices, and interspecies variabilities also exist [48]. Previous scientific studies have compared Doppler ultrasound with increased serum creatinine [49], a situation in which there is already renal functional impairment leading to hemodynamic repercussions. Therefore, the early onset of AKI used in the present study should be taken into account.

Deformation elastography has been studied in CKD in both humans and animals, showing good correlation with healthy subjects and usefulness in chronic conditions [50,51]. However, few studies have looked at this method in AKI. In our study, no differences in elastography scores between the groups were found, which makes sense given the minimal kidney damage seen in the histology and the lack of increased stiffness [52]. It is believed that strain elastography may be more useful for detecting fibrosis in chronic cases and could be important for both humans and animals with acutely exacerbated CKD.

Serum creatinine values were statistically different at the 72 h experimental time point for the GS compared to the CG group; however, none of the animals, regardless of group or analysis period, demonstrated values above the reference range for the studied species [53], aiming to assess early toxicity prior to uremia. We believe that the degree of AKI observed in the animals in this study was insufficient to lower the glomerular filtration rate to the extent that would trigger an increase in SDMA levels, which can rise prior to changes in creatinine and urea [7].

The current study differs from other studies in the scientific literature that used high doses of cyclophosphamide as a nephrotoxic agent. Our aim was to characterize non-fulminant AKI so that parameters of greater early detection could be evaluated and compared with the histopathological gold standard. The number of rodents standardized per group was based on the number of humans affected by acute renal failure [54,55] using a reliability of 90% and a margin of error of 10–20% (www.solvis.com.br).

A limitation of our study is that we did not employ a model of severe nephrotoxicity to induce azotemia. However, our primary objective was to assess early nephrotoxicity via imaging, since late-stage kidney damage is well documented through blood biochemistry. Future research could investigate higher doses or different causes of AKI to better understand mortality differences and improve ultrasound diagnostic accuracy. Additionally, we acknowledge that ultrasound and elastography are operator-dependent techniques. To address this, all assessments were carried out by the same blinded operator, ensuring consistent results and enhancing the reliability of our findings. We emphasize that our results should be interpreted within the specific context of toxic nephropathy. Additional comparative studies between different causes of AKI would be essential to better define the diagnostic performance of ultrasound parameters in different pathophysiological conditions.

## 5. Conclusions

B-mode ultrasonography was able to denote significant early changes in the corticomedullary ratio and renal size, especially in the width of animals subjected to nephrotoxic doses of cyclophosphamide. Doppler ultrasonography and strain elastography did not indicate early abnormalities associated with AKI.

## Figures and Tables

**Table 1:** Criteria for renal assessment based on B-mode ultrasound.

**Echotexture:**
Homogeneous: score 0
Heterogeneous: score 1
**Echogenicity of different renal regions (medulla and pelvis):**
Anechoic: score 1
Hypoechoic: score 2
Hyperechoic: score 3
**Cortical echogenicity in comparison to the spleen or liver:**
Lower than spleen or liver: score 1
0Equal to spleen or liver: score 2
Higher than spleen or liver: score 3
**Cortico-medullary ratio:**
Normal: score 1
Decreased: score 2
Increased: score 3
**Renal length-to-aortic diameter ratio (unit not applicable)**
**Renal dimensions:**
Length (cm)
Width (cm)
Height (cm)
**Renal surface:**
Regular: score 1
Irregular: score 2

## Data Availability

The datasets generated and/or analyzed during this study are included in this published article (and its Supplementary Information) and are available from the corresponding author upon reasonable request.

## Supplementary Materials

The following supporting information can be downloaded at: https://www.mdpi.com/article/10.3390/diagnostics15162034/s1, File S1: Pattern of blood flow and renal resistive index, by the doppler flowmetry analysis, in differents experimental time (24, 48 and 72 h) in animals treated with cyclophosphamide (GC); File S2: Values of creatinine, urea, and SDMA found in animals treated with cyclophosphamide (GC) and 14 saline solution (GS) at different experimental times (24, 48, and 72 h).
