# Supplementary material for: B-Mode Ultrasound May Be an Early Marker in Acute Kidney Injury
Source: Diagnostics (Basel). 2025 Aug 14;15(16):2034. doi: 10.3390/diagnostics15162034 (PMC12385966; doi:10.3390/diagnostics15162034)
Supplement: Supplementary file 1 [file diagnostics-15-02034-s001.zip › diagnostics-3685575-supplementary.pdf]

Supplementary Material File S1

Pattern of blood flow and renal resistive index, by the doppler flowmetry analysis, in differents experimental time (24, 48 and 72 hours) in animals treated with cyclophosphamide (GC).			
**Animal/ Experimental time (hours)**	**Systolic velocity (cm/s)<br><br>left/right kidney**	**Diastolic velocity (cm/s)<br><br>left/right kidney**	**Resistive index<br><br>left/right kidney**
01/24h	8.7/12.3	5.5/5.9	0.37/0.52
02/24h	19.4/10.5	11.6/4.4	0.40/0.58
03/24h	18.2/27.2	6.4/10.7	0.64/0.61
04/24h	13.0/11.2	6.2/9.4	0.52/0.16
05/24h	18.3/14.4	7.6/8.7	0.58/0.4
06/24h	14.6/13.0	7.4/6.9	0.49/0.47
07/24h	21.5/17.3	12.3/9.4	0.43/0.45
01/48h	15.5/10.1	8.7/5.9	0.44/0.42
02/48h	16.9/22.6	8.4/8.4	0.51/0.63
03/48h	22.6/21.9	10.5/11.6	0.54/0.47
04/48h	14.8/19.0	7.3/9.1/	0.51/0.52
05/48h	16.9/12.6	9.8/6.9	0.42/0.45
06/48h	14.4/8.7	11.2/7.3	0.22/0.16
07/48h	22.6/14.1	10.5/11.6	0.54/0.18
01/72h	15.5/18.0	7.6/6.2	0.50/0.65
02/72h	17.6/18.7	7.3/9.4	0.59/0.50
03/72h	18.3/19.0	8.7/8.0	0.52/0.58
04/72h	22.6/20.5	10.5/6.6	0.54/0.68
05/72h	15.5/13.3	7.6/7.6	0.51/0.43
06/72h	19.0/12.6	7.3/7.3	0.62/0.42
07/72h	19.7/14.8	11.6/6.9	0.41/0.53

Supplementary Material File S2

Values of creatinine, urea, and SDMA found in animals treated with cyclophosphamide (GC) and saline solution (GS) at different experimental times (24, 48, and 72 hours).

Creatinine		
**Times experimental**	**GC**	**GS**
**24 hours**	0.4	0.4
	0.5	0.4
	0.6	0.4
	0.7	-
**48 hours**	0.5	0.5
	0.3	0.2
	0.2	0.5
	0.3	
**72 hours**	0.4	0.5
	0.3	0.5
	0.3	0.5
	0.3	-
**Urea**		
**Times experimental**	**GC**	**GS**
**24 hours**	90.0	44.0
	78.0	46.0
	59.0	47.0
	49.0	-
**48 hours**	38.0	36.0
	41.0	48.0
	49.0	40.0
	44.0	-
**72 hours**	59.0	45.0
	56.0	51.0
	35.0	81.0
	51.0	-
**SDMA**		
**Times experimental**	**GC**	**GS**
**24 hours**	14.5	14.9
	18.3	9.0
	9.0	10.5
	9.0	-
**48 hours**	9.0	9.0
	9.0	10.5
	9.0	9.0
	9.0	-
**72 hours**	9.0	10.3
	9.0	10.8

	9.0	13.8
	9.0	-
